# Nutrition, Metabolism, and Physiology in Aquatic Animals: Current Advances and Future Directions

**DOI:** 10.3390/metabo16050306

**Published:** 2026-04-30

**Authors:** Xueshan Li, Ren-Lei Ji

**Affiliations:** 1 State Key Laboratory of Mariculture Breeding, Fisheries College, Jimei University, Xiamen 361021, China; xsli@jmu.edu.cn; 2 Xiamen Key Laboratory for Feed Quality Testing and Safety Evaluation, Fisheries College, Jimei University, Xiamen 361021, China; 3 Department of Biological Chemistry and Molecular Pharmacology, Harvard Medical School, Boston, MA 02215, USA

Aquaculture nutrition research is undergoing a substantive conceptual transition. The field is no longer defined primarily by dietary requirement estimation or by growth-centered feeding trials alone. Instead, current progress increasingly depends on an integrative framework in which nutrients, metabolites, endocrine signals, immune competence, and environmental stressors are interpreted as components of a unified physiological system. Recent reviews have highlighted this shift, particularly the expanding importance of omics approaches, nutrient-sensing pathways, microbiota-dependent metabolism, and metabolism-centered quality regulation in aquatic animals [1,2,3,4,5,6,7]. Within this emerging framework, nutrition can no longer be regarded as a matter of input formulation alone; it must be understood as a regulatory force that shapes growth, metabolic allocation, stress resilience, and health status across tissues and developmental stages [2,3,8,9].

This Special Issue was organized in response to that transition. Its premise is straightforward but consequential: nutrition, metabolism, and physiology in aquatic animals must be studied not as isolated domains, but as an integrated regulatory continuum connecting nutrient acquisition, metabolic remodeling, environmental adaptation, and organismal performance. The contributions included here collectively show that the field is moving beyond descriptive profiling toward a more mechanistically disciplined understanding of metabolic phenotype. At the same time, they also clarify where the discipline remains underdeveloped: causal inference is still limited, cross-tissue integration remains insufficient, and the translation of mechanistic insights into practical feed design and production systems has not yet advanced as far as analytical discovery.

A major theme emerging from this collection is that growth heterogeneity is fundamentally a metabolic and neuroendocrine problem, rather than merely a production irregularity. One contribution uses combined transcriptomics and metabolomics to demonstrate that, under low-phosphorus feeding, fast-growing spotted seabass display stronger hepatic antioxidant capacity, enhanced substance transport, and increased amino acid-related metabolic activity, indicating that differential growth under nutrient constraint reflects distinct adaptive metabolic states rather than simple phenotypic divergence (Contribution 1). A second study, focused on growth-retarded Japanese eel, further strengthens this point by showing that severe growth suppression is associated with lower growth hormone expression, impaired glucose and lipid metabolism, reduced digestive and antioxidant capacity, and a coordinated shift toward anorexigenic signaling (Contribution 2). Taken together, these studies argue that size variation in aquaculture populations should be interpreted as an integrated phenotype emerging from nutrient processing, endocrine regulation, redox state, and appetite control. This conclusion is fully consistent with broader work showing that differential growth in fish is tightly linked to metabolic performance, endocrine signaling, and feeding-related neurophysiology [8,9,10].

The second theme is that feed formulation must be interpreted within metabolic context, not ingredient context alone. One study in this Issue demonstrates, under biofloc culture conditions, that carbohydrate utilization in *Litopenaeus vannamei* is strongly conditioned by the production system itself: an intermediate carbohydrate range supports superior growth and feed efficiency, whereas excessive carbohydrate still imposes oxidative cost even within a microbially buffered environment (Contribution 3). Another contribution shows that, in large yellow croaker, a high dietary palmitic acid to DHA + EPA ratio disrupts glucolipid homeostasis through endocrine and transcriptional regulation, reducing glycogen storage, increasing hepatic lipid accumulation, and altering insulin-, leptin-, and adiponectin-related signaling (Contribution 4). These findings are conceptually important seeing as they challenge the reduction of feed evaluation to proximate composition or ingredient replacement alone. The central issue is not simply how much carbohydrate or lipid is supplied, but how nutrient architecture reshapes systemic metabolic homeostasis. This logic is closely aligned with recent reviews emphasizing that modern aquaculture nutrition must increasingly account for nutrient-sensing pathways, lipid quality, metabolic flexibility, and environmental interaction, rather than relying solely on conventional formulation metrics [2,3,5].

When it comes to the third theme, it extends nutritional physiology into the domain of functional nutrient delivery and metabolic programming in live-feed systems. One contribution shows that β-carotene enrichment in *Artemia* metanauplii significantly alters carotenoid composition and induces transcriptomic changes related to carotenoid uptake, transport, oxidative phosphorylation, and antioxidant defense. Importantly, this study does more than document carotenoid accumulation—it shows that live-feed enrichment can be conceptualized as a mechanistic model of nutrient bioencapsulation, metabolic conversion, and stage-specific functional nutrition (Contribution 5). This is especially relevant because larval and early post-larval phases remain highly sensitive to nutritional inadequacy, and *Artemia* continues to occupy a central position in hatchery nutrition. In this respect, the contribution also connects directly with the broader literature showing that carotenoids are not merely pigmentation factors, but regulators of antioxidant capacity, immune status, growth performance, and developmental quality in aquaculture species [11,12].

The fourth theme is that environmental stress must be interpreted through metabolic control architecture. One paper in this Special Issue demonstrates that UCP1 contributes to acute cold-stress resistance in Nile tilapia through regulation of liver metabolism, mitochondrial membrane potential, and stress hormone-associated adaptation (Contribution 6). This study is valuable not only because it identifies a metabolic effector in thermal resilience, but because it places stress adaptation within a mechanistic framework centered on endocrine signaling and mitochondrial function. Environmental challenges in aquaculture, such as whether thermal, oxidative, osmotic, or hypoxic, cannot be treated as an external disturbance superimposed upon nutritional biology. It is itself a metabolic event. Resilience, therefore, is best understood as a function of regulatory integration rather than tolerance alone. This view is increasingly supported by contemporary work on nutrient sensing, metabolic remodeling, and physiological adaptation in aquatic animals [2,4,5,7].

As for the fifth theme, it concerns the maturation of analytical metabolomics from signal generation to reproducible interpretation. One of the studies in this collection presents a curated biphasic ^1^H NMR workflow for profiling polar metabolites and lipid motifs in sea urchin gonads, thereby producing not merely a dataset-specific signature, but a reusable analytical and annotation framework (Contribution 7). This contribution is notable because it addresses a long-standing limitation of aquatic metabolomics: the generation of biologically interesting chemical profiles without sufficient annotation discipline, transferability, or reporting rigor. The field has now reached a point at which analytical sensitivity alone is no longer the main bottleneck. Increasingly, what is required is reproducible metabolite assignment, transparent confidence grading, and cross-study comparability. That requirement is entirely consistent with recent evaluations of omics in aquatic nutrition, which argue that future value lies not in ever-expanding data production, but in improved integration of acquisition, annotation, and mechanistic interpretation [4].

The conceptual horizon of the Special Issue is extended still further by the review on metabolite-activated GPCRs in teleost physiology and aquaculture development (Contribution 8). By organizing current evidence around succinate-, amino acid-, and lactate-responsive receptors, this article argues that metabolites should be understood not only as nutrients or end products, but also as extracellular signals capable of governing glucose utilization, immune regulation, energy balance, and stress coping. This perspective is especially important because it narrows the conceptual distance between nutrition and physiology. Once metabolites are treated as signaling entities, nutritional physiology can no longer be limited to digestion, absorption, and storage. It must also encompass sensing, receptor-mediated signaling, and downstream regulatory integration. That reframing aligns closely with current work on the microbiota–host axis, which increasingly indicates that diet-derived and microbiota-derived metabolites participate directly in host metabolic and immune regulation [1,4].

The contributions to this Special Issue support three broader conclusions. First, aquatic metabolic research is shifting from descriptive phenotyping to mechanism-aware biology. Second, nutrition, physiology, and environmental response must now be interpreted as an integrated regulatory system rather than as separable research domains. Third, the major unresolved bottleneck is no longer metabolite discovery itself, but the capacity to move from association to causality, from tissue-specific signatures to cross-system integration, and from analytical sophistication to translational utility. These conclusions are strongly reinforced by recent reviews of the field, which identify omics integration, microbiota-dependent metabolism, mTOR-centered nutrient sensing, and dynamic metabolic remodeling as critical frontiers in aquatic animal nutrition and physiology [1,2,3,4,5].

This collection also brings current limitations into sharper focus. Many studies remain cross-sectional, which is highly useful for pathway discovery but less powerful for resolving temporal sequence and adaptive trajectory. Functional validation remains uneven, especially for candidate metabolites, receptors, transporters, and transcriptional regulators. Cross-tissue integration remains less developed than single-organ analysis, despite the fact that appetite, hepatic metabolism, immune state, and environmental adaptation are necessarily systemic. Finally, the link between mechanistic discovery and production-level application remains underdeveloped. Precision aquaculture will depend not only on identifying biomarkers, but on determining whether those biomarkers are causal, how stable they are across environmental contexts, and whether they can be translated into feed design, live-feed optimization, environmental management, or selective breeding.

For that reason, several priorities should guide the next phase of research. First, longitudinal and perturbation-based designs are needed to resolve metabolic trajectories rather than static contrasts. Second, studies addressing growth, stress, and nutrient allocation should increasingly adopt multi-tissue approaches. Third, microbiota-derived metabolites and receptor-mediated nutrient sensing deserve substantially greater attention, especially in the context of functional feeds and system-level metabolic regulation. Fourth, metabolomics workflows in aquatic systems require stronger standardization in annotation, reporting, and validation. Finally, translational pipelines must be strengthened so that metabolic knowledge can inform farm-relevant interventions, including feed optimization, resilience-oriented production strategies, and biomarker-assisted breeding. The long-term significance of metabolic physiology in aquaculture will depend on whether it can move from explanatory science to decision-grade biology.

In summary, this Special Issue reflects the increasing maturity of research at the interface of nutrition, metabolism, and physiology in aquatic animals. The eight contributions collectively show that metabolic biology is now central to major aquaculture questions, including growth heterogeneity, nutrient utilization, stress adaptation, functional feed design, analytical rigor, and metabolite signaling. We hope that this collection will stimulate further work that is not only technically sophisticated, but also conceptually integrated, mechanistically grounded, and translationally relevant.

## Data Availability

No new data were created or analyzed in this study. Data sharing is not applicable to this article.
